# What Does the *n*-Back Task Measure as We Get Older? Relations Between Working-Memory Measures and Other Cognitive Functions Across the Lifespan

**DOI:** 10.3389/fpsyg.2018.02208

**Published:** 2018-11-26

**Authors:** Patrick D. Gajewski, Eva Hanisch, Michael Falkenstein, Sven Thönes, Edmund Wascher

**Affiliations:** ^1^Department of Ergonomics, Leibniz Research Centre for Working Environment and Human Factors, Technical University of Dortmund, Dortmund, Germany; ^2^Department of Psychology, Technical University of Dortmund, Dortmund, Germany; ^3^Institute for Working, Learning and Aging, Bochum, Germany

**Keywords:** aging, working memory, *n-*back, cognitive functions, stroop interference, attention, verbal memory

## Abstract

Working memory (WM) declines with increasing age. The WM capacity is often measured by means of the computerized version of the *n*-back task. Although the *n*-back task is widely used in aging research, little is known about its construct validity and specific cognitive functions involved in this task. Moreover, to date, no studies analyzed the construct validity as a function of age. To this end, we conducted a study in a sample of *N* = 533 individuals aged between 20 and 80 years. The sample was divided into three age groups: young (20–40), middle-aged (41–60), and old (61–80 years). A number of psychometric tests was selected that measure attention, memory, and executive control to elucidate the impact of these constructs on *n*-back performance. A series of correlation analyses was conducted to assess the relationship between *n*-back performance and specific cognitive functions in each age group separately. The results show a progressive increase in reaction times and a decrease in the proportion of detected targets from young to old subjects. Age-related impairments were also found in all psychometric tests except for the vocabulary choice test measuring crystallized intelligence. Most importantly, correlations yielded different age-related patterns of functions contributing to performance in the *n*-back task: whereas performance was most related to executive functions in young age, a combination of attentional and executive processes was associated with performance in middle-aged subjects. In contrast, in older age, mainly attentional, verbal memory, and updating and to a lesser extent executive processes seem to play a crucial role in the *n*-back task, suggesting a shift of processing strategies across the lifespan.

## Introduction

Working memory (WM) is a complex system, in which incoming information is maintained and processed despite interference and distraction (Miyake, 2001; Conway et al., 2005; Diamond, 2013). WM stores and updates relevant information to enable goal directed behavior. Older theories base on Baddeley’s (1986) account, which defines WM as at least two slave systems (the phonological loop and the visuo-spatial sketchpad). These systems maintain incoming information and are being controlled by an amodal central executive. It is assumed that the visuo-spatial sketchpad is involved in setting up and maintaining visuospatial information, while the phonological loop represents a temporary storage for speech-based information. The central executive controls and coordinates the slave systems.

Different tasks require more or less activation of the central executive. There are situations in which only short-term memory capacity (STMC), a domain-specific skill is challenged, for example when we need to keep a telephone number in mind. Information has to be stored but not manipulated. Executive attention is required when we need to process additional information simultaneously (Engle et al., 1999; Unsworth and Engle, 2007; Myers et al., 2017). Kane and Engle (2002) elaborated on the question what abilities are asked for in interference-free and interference-rich conditions. According to them, ‘executive attention’ is only required when information has to be maintained during interference. Otherwise task-relevant information can be retrieved from the long-term storage. This model is consistent with common structural (Baddeley, 1986) as well as functional models of storage (Nairne, 2002).

However, more recent models questioned the existence of the central executive and provided a functional explanation of processes involved in WM. The crucial functions are updating, i.e., the ability to replace stored information by new upcoming information (Ecker et al., 2014; Rey-Mermet et al., 2017) and maintenance of the stored unit in stable manner, impenetrable to irrelevant distraction from the environment. Updating and maintenance are in *flexibility* vs. *stability* conflict as the new information can be relevant and trigger updating or can be irrelevant and should be inhibited. Thus, a control mechanism is required to regulate the two functions (Rac-Lubashevsky and Kessler, 2016a,b). Recent WM theories replaced the controlling instance by an input-gating mechanism. This mechanism shields the maintained information and enables stability by closing the gate whereas opening of the gate reflects updating of new relevant information (Kessler and Oberauer, 2014, 2015; Chatham and Badre, 2015). On the neurobiological level, the gating process was assumed to accrue from a dynamic regulation of neuronal transmission between prefrontal cortex and basal ganglia by dopamine release (Miller and Cohen, 2001; Hazy et al., 2006; O’Reilly and Frank, 2006). These functions and the related WM performance can be improved by cognitive training in young (Jaeggi et al., 2008) as well as old individuals (Karbach and Verhaeghen, 2014). These effects are accompanied by changes in electrophysiological activity in frontal brain areas (Gajewski and Falkenstein, 2018).

### Working Memory Capacity, Short-Term Memory Capacity, and Age

Executive functions decline with increasing age (Salthouse, 1991, 2015; Van der Linden et al., 1994; Grégoire and Van der Linden, 1997; Braver and West, 2008; Basak and Verhaeghen, 2011; Gajewski et al., 2018). However, recent meta-analytical results question the generalizability of this statement (Verhaeghen, 2011, 2014; Rey-Mermet and Gade, 2017).

It has been frequently shown that aging is associated with WM decline (Hasher and Zacks, 1988; Braver and West, 2008; Salthouse, 2015). In particular, it was assumed that executive attention is subjected to age-related changes (Salthouse, 1991; Van der Linden et al., 1994). Therefore, simple and complex span tasks have been developed in order to provide differentiated measurements of domain-specific skills and domain-general executive attention (Daneman and Carpenter, 1980; Case et al., 1982; Turner and Engle, 1989; Wilhelm et al., 2013), which is especially interesting with regard to age. Importantly, it has been shown that older participants have more difficulties than young subjects in maintaining information while processing additional information simultaneously (Van der Linden et al., 1994), which suggests impaired executive functions and reduced working memory capacity (WMC) in older subjects. WMC was related to individual differences in the limited capacity of a person’s WM and was usually assessed by means of complex span paradigms. However, more recent studies extracted further indicators of WMC, such as the ability to build, maintain, and update arbitrary bindings (Wilhelm et al., 2013).

It has been assumed that STMC is less vulnerable to age than WMC (Craik, 1977; Welford, 1980; Van der Linden et al., 1994). However, employing the Forward–Backward-Digit-Span-Task, it has been shown that elderly participants perform worse in both, the Forward- and the Backward-condition (Grégoire and Van der Linden, 1997). Therefore, also STM span seems to be affected by age-related decline.

### Working Memory and the *n*-Back Task

Age-related changes of WM capacity were reported in several studies using different WM-tasks (Hedden and Gabrieli, 2004; Salthouse, 2015 for reviews). A common paradigm to assess WMC is the so-called *n*-back task (Kirchner, 1958). In the *n*-back task participants are presented a series of visual stimuli. They are asked for each stimulus whether it matches a stimulus *n* trials before. For example, in a 2-back task, in which the trials consist of letters, participants have to decide whether the current letter is the same as the letter in trial *n* – 2. The task requires a cascade of cognitive processes: the task requires encoding and a temporary storage of each stimulus *n* of the stimulus sequence in WM and a continuous updating of incoming stimuli. At the same time, irrelevant items have to be inhibited and the currently irrelevant items abandoned from WM. A counting and matching process between the upcoming and stored stimulus in WM is necessary to make the decision whether the stimuli are the same to initiate a correct response (Rac-Lubashevsky and Kessler, 2016a). This complexity of involved cognitive sub-processes makes it difficult to extract the crucial mechanism contributing to the age-related decline of *n*-back performance.

The *n*-back task has face validity as a WM task since it seems to require maintaining, continuous updating and processing of information. Since at least two tasks, maintaining and manipulating information, have to be processed simultaneously, it apparently matches the criteria of domain-general executive attention (Kane and Engle, 2002; Kane et al., 2004; Wilhelm et al., 2013). However, the *n*-back paradigm has recently become the focus of doubts concerning its construct validity as a WM task (Kane et al., 2007; Miller et al., 2009; Jaeggi et al., 2010). Although the *n*-back task exists since 1958, little is known about its psychometric properties.

Aging effects in this task have been reported repeatedly (Oberauer, 2005; Verhaeghen and Basak, 2005; Basak and Verhaeghen, 2011, see also Bopp and Verhaeghen, 2018 for a recent meta-analysis). The use of the *n*-back task has increased with rising interest in studies using neuroscientific methods like functional magnetic resonance imaging (fMRI) and event-related potentials (ERPs). Presuming that *n*-back requires specific functions that are believed to represent the functionality of WM, such as updating and maintenance, it has been deployed widely in neuroimaging studies also in the context of aging (Jonides et al., 1997; Missonnier et al., 2004; Owen et al., 2005; Daffner et al., 2011; Wild-Wall et al., 2011; Gajewski and Falkenstein, 2014, 2018). Therefore, it is important to note that the construct validity of the *n*-back task has not been analyzed sufficiently yet. If we cannot rely on *n*-back as a WM task, we cannot rely on inferences drawn about WM on a neuroscientific level in a study in which *n*-back has been used. Especially with regard to age-related changes, it would be premature to assume that impairments in elderly subjects are associated with decreased functionality of domain-general executive attention of WM if *n*-back is not an appropriate instrument for measuring WMC. More plausible is, however, that WM may reflect a conglomerate of basic psychological constructs like attention, updating, and executive functions. In order to answer the question which changes are to be expected with increasing age it is of utmost importance that one can rely on valid instruments. Thus, the aim of the present study is to replicate previous findings regarding decline of WM across the lifespan using the *n*-back task in a large sample of participants and to extract the crucial psychological constructs involved in this performance decline.

### Findings on the Construct Validity of *n*-Back

The ambiguous results from the little research in this area raise even more questions whether the *n*-back task measures WMC or shares variance with other constructs such as selective attention, stimulus updating or interference processing. A few studies addressed this question by correlating *n*-back with other measures (e.g., Kane et al., 2007; Miller et al., 2009; Schmiedek et al., 2014).

### *n*-Back and Other WMC Measures

Studies in which *n-*back has been correlated with WMC measures such as reading span tasks or operation span tasks revealed rather weak correlations (ranging between *r* = 0.10 and *r* = 0.24; Roberts and Gibson, 2002; Oberauer et al., 2003, 2005; Kane et al., 2007; Colom et al., 2008; Unsworth, 2010). In these studies, only single reading or operation span tasks were correlated with *n*-back. Positive findings were those of Shamosh et al. (2008) who employed a composite score of four complex span measures (operation span, reading span, symmetry span, rotation span) and achieved a correlation with *n*-back of *r* = 0.55. Two further studies (Shelton et al., 2007, 2009) revealed a correlation of *r* = 0.46 between operation span and a composite *n*-back score (0-, 1-, 2-, and 3-back). Schmiedek et al. (2014) found correlations between *r* = 0.31 and *r* = 0.69 in young and *r* = 0.42 and *r* = 0.66 in old subjects for numerical *n-*back and reading span, counting span, rotation span, *n*-back spatial, memory updating numerical, memory updating spatial, alpha span, and animal span.

### *n*-Back and STM Measures

Findings that speak against *n*-back validity as a measure of WMC are those that yield stronger correlations between *n*-back and STMC tasks than between *n*-back and WMC tasks (correlations between *r* = 0.12 and *r* = 0.53; Dobbs and Rule, 1989; Gevins and Smith, 2000; Roberts and Gibson, 2002; Oberauer, 2005; Shelton et al., 2007, 2009; Colom et al., 2008).

### *n*-Back and the Stroop Task

Interestingly, another study has shown that results from an *n*-back task share more variance with the performance in a Stroop task than they do with a STM span task (Kwong See and Ryan, 1995). A study conducted with children (Ciesielski et al., 2006) also revealed that 2-back performance is substantially correlated with Stroop performance (*r* = 0.55) and verbal fluency (*r* = 0.59). There were other studies which provided only weak correlations *r* = 0.10 between 2-back and Stroop performance (Friedman et al., 2006, 2008). Miller et al. (2009) reported *r* = 0.26 for the association between Stroop color naming and 2-back in speed and *r* = 0.43 in accuracy.

### *n*-Back and Measures of Fluid Intelligence

Updating WM with new information is substantial for high-level cognition, such as arithmetic operation, comprehension, and reasoning (e.g., Rac-Lubashevsky and Kessler, 2016b). Thus, it can be expected that WM shares considerable variance with measures of fluid intelligence (Gf) (Kyllonen and Christal, 1990; Conway et al., 2003; Ackerman et al., 2005; Kane et al., 2004, 2005; Oberauer et al., 2005). Some studies reported correlations between *n*-back performance and various intelligence measures (Gevins and Smith, 2000; Friedman et al., 2006, 2008; Van Leeuwen et al., 2007; Salthouse et al., 2008; Shelton et al., 2009; Waiter et al., 2009). It has been shown that 2-back latencies decrease with increasing IQ levels (Gevins and Smith, 2000; Hockey and Geffen, 2004). Engle et al. (1999) found that *n*-back is strongly connected to fluid intelligence but not to STM span. Kane et al. (2007) presented an *n*-back-study that also included two tests of WM span and general fluid intelligence. WM span and *n*-back correlated weakly and both accounted for independent variance in general fluid intelligence. It has been concluded that *n*-back reflects a construct different from that of WM span. Similar results were obtained by Miller et al. (2009).

### The Present Study

Previous research reported above evaluated a general association between *n*-back as measure of WMC and basic psychological constructs regardless of age. The present study aims to fill the gap. Thus, we conducted a study investigating specific mechanisms underlying WM decline across the life span. To this end, a large sample of participants conducted the *n*-back task and was divided into three age groups: young, middle-aged, and old individuals. Whereas most studies contrasted performance between young and old participants, the middle-aged group was often neglected but provides important information about the beginning of the age-related decline in different cognitive domains. Furthermore, we used a number of psychometric tests that cover a wide range of psychological constructs like selective and sustained attention, updating, different aspects of memory, such as short- and long-term memory, WM, verbal fluency, crystallized intelligence as well as executive control (interference control, and task switching) to elucidate the association between these constructs and *n*-back performance as a function of age. We conducted correlation analyses assessing the relationship between *n*-back performance and the psychological functions in each group separately to understand age-related WM decline in more detail.

First, in accordance with previous findings on age-associated cognitive impairments, we hypothesize that fluid cognitive functions like attention, memory and executive control decline as a function of age (Salthouse, 1991, 2015; Van der Linden et al., 1994; Grégoire and Van der Linden, 1997; Braver and West, 2008; Basak and Verhaeghen, 2011). In contrast, crystallized functions (Horn and Cattell, 1967) should not suffer from age (Baltes, 1987). Second, we assume that age-related effects are not due to a general slowing in older age (Salthouse, 2000). We analyze specific decline in executive functions independently of general speed of processing by computing difference scores between conditions involving and not-involving executive control that eliminate individual RT differences (e.g., 2-back–0-back, Stroop 3–Stroop 2, TMT B–TMT A).

Third, we expect that the age-related reduction of WM performance as reflected in the *n*-back task cannot be explained by an impairment of a unitary WM function. Instead, we expect that performance in the *n*-back task is associated with different cognitive mechanisms depending on age, suggesting an involuntary shift of strategy with age to successfully perform the WM task. We hypothesize that while young individuals rely on executive processes to resist interference from concurrent items, older ones involve primarily attentional resources and mnemonic functions to overcome lapses in executive control. Middle-aged participants are expected to show a mixed pattern of results.

## Materials and Methods

### Participants

The data for the present study have been collected in multiple studies: pre-tests of two training studies with old (*n* = 152; Gajewski and Falkenstein, 2012, 2018) and middle-aged participants (*n* = 58; Gajewski et al., 2017), a study with physically active elderly (*n* = 21; Gajewski and Falkenstein, 2015), a study including young participants (*n* = 36; Gajewski and Falkenstein, 2014), and an ongoing study including subjects aged between 20 and 70 years that aims at analysing effects of biological and environmental factors on cognitive aging in a longitudinal design (Dortmund Vital Study; *n* = 266).

A total of 533 healthy subjects without neurological or psychiatric impairments participated in the present study and completed the *n-*back task. Due to some drop out in single tests, the total number of subjects that have completed a particular test varied between *n* = 420 and *n* = 533. Four hundred and twenty subjects completed all psychometric tests and provided the data for the explorative correlation analysis. The participants were between 20 and 80 years old. The sample was divided into three groups by age. The *young group* consisted of 157 participants [20–40 years of age; *M* = 29.1; *SD* = 5.4; 66 males (42%), 91 females (58%)], the *middle-aged group* consisted of 182 participants [41–60 years of age; *M* = 49.4; *SD* = 5.0; 90 males (49%), 92 females (51%)] and the old group consisted of 194 participants [61–80 years of age; *M* = 70.0; *SD* = 4.9; 93 males (48%), 100 females (52%)]. All subjects had normal or corrected-to-normal vision. Educational level differed between age groups [*F*(2,529) = 59.6, *p* < 0.0001] due to historical reasons and changes in education policy across decades. In particular, the older group had lower education [mainly elementary school (8th grade) and less often grammar school]. In contrast, the young group’s education was at least intermediate secondary school (10th grade).

All experiments, in which the data were collected, were reviewed and approved by the ethics committee of the Leibniz Research Centre of Working Environment and Human Factors, Dortmund, Germany. All subjects gave written informed consent in accordance with the Declaration of Helsinki.

### Apparatus and Procedure for the *n*-Back Task

Participants were seated comfortably in front of a monitor (17 in., refresh rate: 100 Hz, resolution: 640 × 480 pixels). The distance between the eyes and the monitor was approximately 70 cm. The letters were presented within a 16 × 16 mm matrix in the middle of the monitor (1.6° matrix/eye). Each letter was centrally adjusted. A checkpoint (5 × 5 mm, 0.5° checkpoint/eye) was presented before each stimulus, which was also located in the center of the monitor. The interstimulus interval (ISI-time) was set to 1,500 ms. Maximum reaction time (RT) of 1,200 ms and a minimum RT of 100 ms were allowed. Premature and late responses were categorized as missings. Two blocks were applied. The 0-back block (two-alternative forced choice task) served as a control condition with low WM demands. This block consisted of 102 trials. Participants were asked to respond to the occurrence of each letter ‘X’ by pressing a key with the index finger of the right hand. The task in the second block (2-back condition) demanded WM capacity. In the 2-back-condition (i.e., experimental condition), participants were asked to decide for each stimulus whether it matches the second last one, again by pressing the designated key. Otherwise no response was required. The 2-back-condition consisted of 156 trials. Each block consisted of 20% target and 80% non-target letters. RT and missings were analyzed for each block. The two blocks were presented without a break. Each participant received the same random series of letters. Each stimulus was presented for 300 ms regardless of whether the participant pressed a key or not.

### Psychometric Tests

#### The Forward/Backward-Digit-Span-Task

In the Forward/Backward-Digit-Span-Task (‘Forward/ Backward-DS,’ from NAI, Oswald and Fleischmann, 1986) a sequence of digits was verbally presented to the participant (one digit per second). After the full presentation of a sequence, the participant’s task was to repeat the full sequence exactly as it has been presented in the Forward-condition and in reverse order in the Backward-condition. The digit sequences consisted of three to eight digits and were presented in ascending order. If a sequence, of three digits for example, was reproduced correctly, the participant was given the next larger sequence (e.g., of four digits). If a sequence was reproduced incorrectly, the participant was given a second sequence of equal length. If this second sequence was also reproduced incorrectly, the investigator stopped the procedure and moved on to the next block (Backward-DS). The number of correctly repeated sequences represents the score of interest (dependent measure) of the test. The test is considered to measure maintenance and recall of information, i.e., short-term memory (Forward-DS), and flexible processing of information stored in WM (Backward-DS).

#### The Word Fluency Test

In the Word Fluency Test (from LPS, Horn, 1983), participants were asked to recall as many words beginning with a specific letter as they could think of within a given time. Three trials were conducted. In the first trial, participants were asked for words with the initial letter B; in the second trial for words with the initial F and in the third trial for words with the initial L (BFL). Participants were given 30 s for each trial. The produced words were added up and represent the test result (dependent variable). The test measures the ability to access the verbal lexicon, semantic memory, the scope of vocabulary, cognitive flexibility and divergent thinking.

#### Verbal Learning and Memory Test (VLMT)

The Verbal Learning and Memory Test is a German version of the as Rey Auditory Verbal Learning Test (RAVLT; Schmidt, 1996). In the first part, a 15 noun-word list (list A) was read to the participants with a presentation rate of one word per second. After presentation of the words, the subjects were requested to recall as many words as possible. This procedure was repeated five times, and after each trial the number of correctly retrieved words was recorded. To assess the *learning ability*, the number of correctly reproduced items was added up across the five trials, representing the overall score (dependent measure). Subsequently, an interference-list of 15 other nouns (list B) was presented to the participants and they were asked to recall as many list-B words as possible to assess *pro-active inhibition* of the previously learned words. Immediately after recall of list B, the participants were again asked to recall list A (short recall, A6) to evaluate *retro-active inhibition*. *Delayed recall* of list A was measured 30 min after the immediate recall (long recall, A7) (with no other verbal memory tests administered in between). Directly after the long recall, A7, a *recognition trial* of 50 words containing the 15 words from list A and 15 distracter items was applied (10 distracter words were semantically or phonetically similar to the target words). The test measures different aspects of verbal memory.

#### Multiple Choice Vocabulary Test (MWT-B)

The Multiple Choice Vocabulary Test (MWT-B; Lehrl, 1995) measures crystallized intelligence and consists of 37 items each item containing five words. One of them reflects a meaningful word the other verbally similar words are meaningless. The subjects are required to mark the correct word. The difficulty of items increases with increasing item number. The number of correctly identified meaningful words allows assessment of the IQ.

#### Digit-Symbol-Test

The Digit-Symbol-Test is an evaluation tool used to assess cognitive functioning. It initially was part of the Wechsler Adult Intelligence Test (WAIS; Wechsler, 1956). In particular, this test appears to be sensitive to changes in people whose cognition is quite good, whereas other tests might be unable to differentiate between persons with normal cognition and those with just the beginnings of mild cognitive impairment. The Digit-Symbol-Test measures processing speed, WM, visuospatial processing and attention.

#### The d2 Test

In the d2 Test (Brickenkamp, 1972), subjects were given a sheet of paper with 14 lines consisting of 47 letters (d and p) with one to four dashes (‘), located either individually or in pairs above or below the letter. Participants were asked to go as fast as possible through each line and identify every d with two dashes by crossing it out. After 20 s of processing one line, the subjects were told to move on to the next line and to continue. The number of correctly identified d’s with two dashes were added and represents the test score. The d2 Test is a measurement of focussed and sustained attention as well as processing speed. A revised version, d2-R, extending the length of the test lines was released in 2015.

#### The Stroop Task

The Stroop task (from NAI, Oswald and Fleischmann, 1986) consisted of three parts. In the first part (Stroop 1), subjects were given a sheet of paper with a number of names of colors printed in black. The participants were asked to read them out aloud as fast as possible. In the second part (Stroop 2), participants were handed another sheet of paper with colored bars on it. Participants were told to name the colors. In the third condition (Stroop 3), subjects were given a sheet of paper with names of colors printed in various colors, which did not match the names of the colors (e.g., ‘GREEN’ was printed in red color). Subjects had to name the colors the words were printed in as fast as possible. The time participants needed to fulfill each condition was measured. There was the same number of words than of colored bars in each condition. The final time of the third list is considered as an indicator of interference processing and inhibitory control as one of the core executive functions.

In order to further validate the results, we used error rates of a computer-based Stroop task from the block including interference (see Gajewski and Falkenstein, 2015, for details of the task).

#### Trail Making Test (TMT)

The Trail Making Test (TMT) consists of parts A and B. Both parts consist of 25 circles distributed over a sheet of paper. In Part A, the circles are numbered 1–25, and the participant should draw lines to connect the numbers in ascending order. In Part B, the circles include both numbers (1–13) and letters (A–L). As in Part A, the participant draws lines to connect the circles in an ascending pattern, but with the added task of alternating between the numbers and letters (i.e., 1-A-2-B-3-C, etc.). The test is thought to measure speed of processing, focussed attention, task switching and updating, which represent crucial executive functions.

### Statistical Analysis

#### *n*-Back Task

A mixed analysis of variance (mixed ANOVA) was conducted to compare the effect of age (young vs. middle-aged vs. old; between-subjects factor) and task condition (0-back vs. 2-back; within-subject factor) on RT and the number of missings. Significant interactions and group differences were further analyzed using one-way ANOVAs with *post hoc* comparisons using Bonferroni correction. We expected the difference between 0-back and 2-back conditions to be pronounced in older participants, which should be reflected in an interaction between task type and age.

#### Analysis of the Psychometric Tasks

For the analyses of psychometric tests with multiple conditions, such as Forward–Backward-DS, Stroop, and TMT, mixed ANOVAs were conducted to compare the effect of age group and task condition. In the digit-span task, the number of correctly repeated numerical series in the Forward vs. Backward-DS task was analyzed. In the Stroop task, effects of the task type (Stroop 1, Stroop 2, Stroop 3) on the time needed to perform the task was analyzed. To assess interference costs, a difference score between Stroop 3 and Stroop 2 was conducted and evaluated. Similarly, in the TMT task, the time to perform tasks A and B was analyzed. The difference between tasks A and B represents switch costs.

Tasks consisting of only one condition, such as word-fluency, MWT-B, d2, and Digit-Symbol-Test, were analyzed using one-way ANOVAs. Also, the different memory components in the VLMT, like *learning ability* as reflected in the total score of the trials 1 to 5, *pro-active inhibition* measured by the number of correctly named items from list B, *retro-active inhibition* (number of correctly named items from list A after retrieval of the interference list B) and *delayed recall* (number of correctly named items 30 min later, etc., were analyzed using a series of Bonferroni corrected one-way ANOVAs. Specific group differences were evaluated using Bonferroni corrected *post hoc* tests.

As a revised version of the d2 Test (d2-R) which is not directly comparable to the original version was used in a part of the sample, we report *z*-transformed values of the test.

Finally, we report re-test reliability scores (Pearson correlations) of the tests, which reflects the extent to which similar scores are obtained when the scale is administered on different occasions. Re-test reliability was obtained from 141 participants from the oldest and from 58 of the middle-aged groups. The re-tests were conducted as post-measures in the context of two training studies (Gajewski and Falkenstein, 2012; Gajewski et al., 2017).

### Correlation Analyses

As the measures of interest in the correlation analyses we defined the differences in RT and accuracy between the 0-back and 2-back condition, which should reflect the specific WM-related task demands (storage and updating). By means of three correlation analyses (separate analyses for the three age groups), we investigated the relationships between effects of task condition in the *n*-back task (the increase in RT and decrease in accuracy from 0-back to 2-back) and performance in the different psychometric tasks. Note that these analyses were explorative in nature in order to evaluate which processes (attention, inhibition, processing speed, etc.) are best related to *n*-back performance and specific age-related changes. Due to the large sample size (power) and multiple testing, we adjusted the alpha-level to 0.005 and focus on the size of the different correlation coefficients (*r*). This analyses included *n* = 420 subjects.

Additionally we conducted a correlation analysis for the 2-back–0-back difference scores and the difference score incongruent–congruent in accuracy of a computer-based Stroop task for each age group separately. This analyses included *n* = 525 subjects in total.

## Results

### *n*-Back Task

**FIGURE 1:**
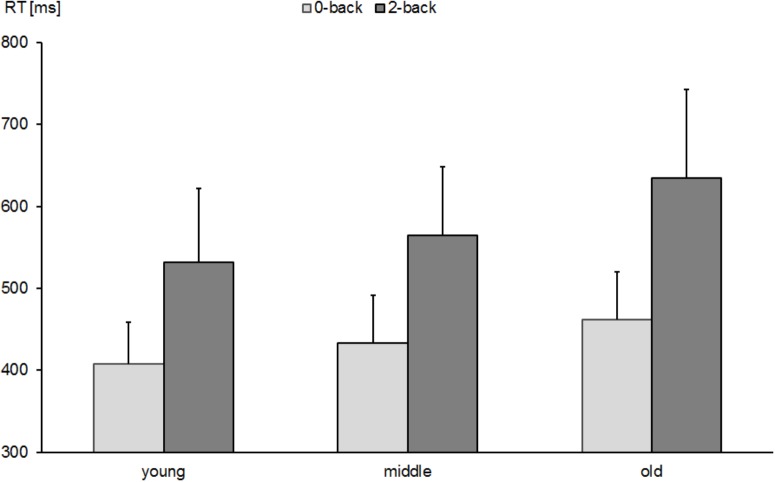
RTs in 0-back- and 2-back tasks in young, middle-aged, and old groups. Error bars reflect standard deviations.

The repeated measures ANOVA indicated main effects of task condition [0-back vs. 2-back; *F*(1,530) = 1590.5, *p* < 0.0001, $\eta_{p}^{2}$ = 0.750] and age group [young vs. middle-aged vs. old; *F*(2,530) = 62.4, *p* < 0.0001, $\eta_{p}^{2}$ = 0.191] on RT as well as an interaction between both factors [*F*(2,530) = 18.9, *p* < 0.0001, $\eta_{p}^{2}$ = 0.067]. This interaction indicated a larger effect of task condition in older than middle-aged and young participants. In order to decompose the interaction, we computed the differences between the 2-back and 0-back condition and compared it between the groups. A one-way ANOVA with *post hoc* comparisons between the three age groups revealed differences between young and old participants (*M*: 123 ms; *SD*: 73 vs. *M*: 172 ms; *SD*: 92, *p* < 0.0001) and between middle-aged and old participants (*M*: 130 ms; *SD*: 76 vs. *M*: 172 ms; *SD*: 92, *p* < 0.0001), while no difference was observed between young the middle-aged participants (*p* > 0.05). Figure 1 shows mean and standard deviations of RTs as a function of age group and task condition.

**FIGURE 2:**
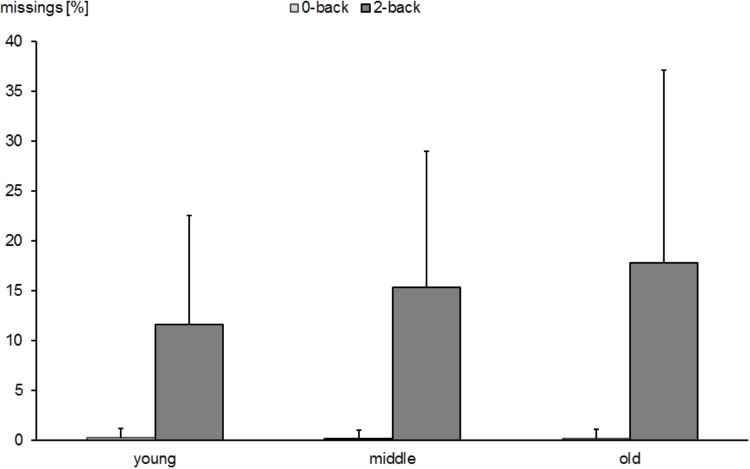
Percent of missed targets in 0-back- and 2-back tasks in young, middle-aged, and old groups. Error bars reflect standard deviations.

A similar pattern was found for the number of missed targets (Figure 2). There were main effects of task condition [*F*(1,530) = 494.4, *p* < 0.0001, $\eta_{p}^{2}$ = 0.483] and age group [*F*(2,530) = 6.9, *p* < 0.001, $\eta_{p}^{2}$ = 0.025] and a significant interaction of the two factors [*F*(2,530) = 7.4, *p* < 0.001, $\eta_{p}^{2}$ = 0.027]. This interaction was due to the older participants showing a higher proportion of missed targets in the 2-back condition (*M* = 17.8%; *SD* = 19.3) than the young subjects did (*M* = 11.5%; *SD* = 10.9; *p* < 0.0001), whereas no group differences were found in the 0-back task. Similar to the RTs, this pattern was corroborated by group differences in the computed difference scores between the 2- and 0-back conditions (old: *M* = 17.6%, *SD* = 19.1 vs. young: *M* = 11.3%, *SD* = 10.9, *p* < 0.0001; old vs. middle-aged: *M* = 15.2%, *SD* = 13.6, *p* > 0.05; and young vs. middle-aged, *p* < 0.05).

### Psychometric Tests

#### Forward and Backward Digit-Span

The ANOVA of the Forward/Backward-DS revealed main effects for task condition [‘Forward’ vs. ‘Backward’; *F*(1,526) = 151.7, *p* < 0.0001, $\eta_{p}^{2}$ = 0.224] and age group [*F*(2,526) = 23.2, *p* < 0.0001, $\eta_{p}^{2}$ = 0.081] and a significant interaction of the two factors [*F*(2,526) = 9.1, *p* < 0.0001, $\eta_{p}^{2}$ = 0.034]. Bonferroni corrected *post hoc* tests revealed that young subjects (*M* = 8.1; *SD* = 2.3) did not repeat more digit series successfully in the Forward-condition compared to old subjects (*M* = 7.6; *SD* = 2.6, *p* > 0.05). However, the middle-aged group (*M* = 8.8; *SD* = 3.0) showed clearly better performance relative to old subjects (*p* < 0.0001). The re-test reliability of the digit span forward test was 0.421 (*p* < 0.005) in middle-aged and 0.564 (*p* < 0.0001) in the older group.

In the Backward-condition, both young and middle-aged participants outperformed older subjects (*M* = 7.4; *SD* = 2.1 vs. *M* = 7.5; *SD* = 2.5; vs. *M* = 5.9; SD = 1.5; both *p* values < 0.0001; for young, middle-aged, and old subjects, respectively). No difference was found between young and middle-aged groups (*p* > 0.05). The re-test reliability of the digit span backward test was 0.345 (*p* < 0.01) in middle-aged and 0.457 (*p* < 0.0001) in the older group.

**FIGURE 3:**
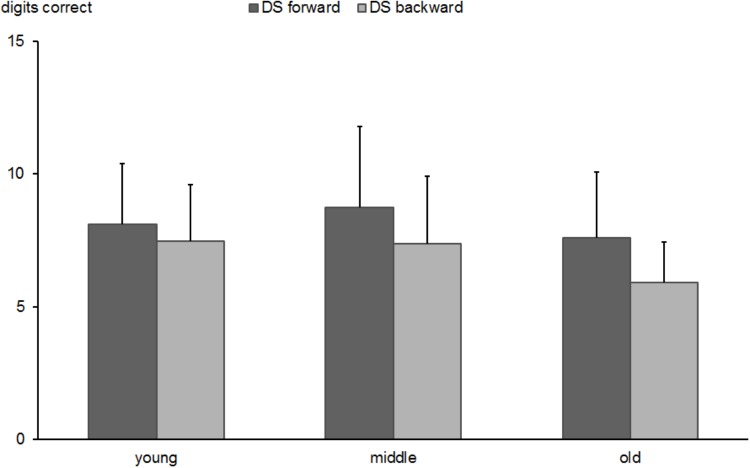
Digit span. Number of correctly repeated numerical series in forward and in reverse order in young, middle-aged, and old groups. Error bars reflect standard deviations.

Figure 3 shows the descriptive results.

#### Verbal Fluency

**FIGURE 4:**
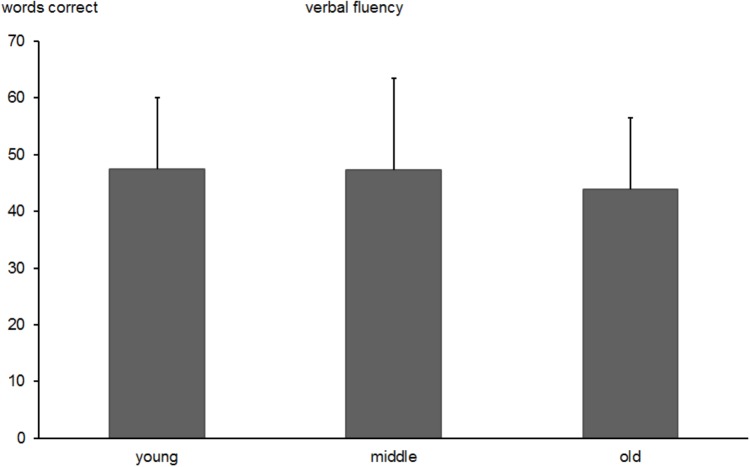
Total number of correctly produced words in the Verbal-Fluency Test in young, middle-aged, and old groups. Error bars reflect standard deviations.

The one-way ANOVA yielded a significant effect of age group [*F*(2,518) = 4.0, *p* < 0.05]. The number of produced words in the older group (*M* = 43.8, *SD* = 12.6) was lower than in middle-aged (*M* = 47.3, *SD* = 16.6; *p* < 0.05) and younger subjects (*M* = 47.5, *SD* = 12.6; *p* < 0.05). The descriptive results are presented in Figure 4. No difference was found between young and middle-aged subjects (*p* > 0.05). The re-test reliability of the verbal fluency test was 0.657 (*p* < 0.0001) in middle-aged and 0.730 (*p* < 0.0001) in the older group.

#### Verbal Learning and Memory Test (VLMT)

**FIGURE 5:**
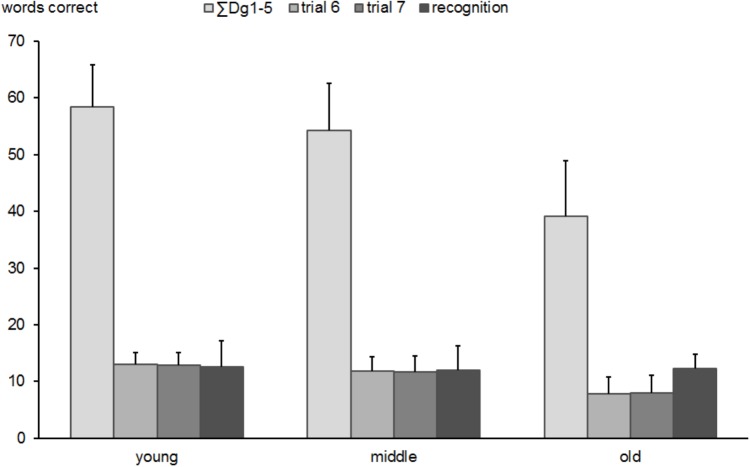
Total number of correctly produced words in subtests of the Verbal Learning and Memory Test (VLMT) in young, middle-aged, and old groups. Error bars reflect standard deviations.

A series of one-way ANOVAs conducted for the most relevant parameters of the VLMT showed reliable group differences (see Figure 5).

The learning performance as reflected by the total score of the trials 1 to 5 showed an age-related decrease [*F*(2,518) = 243.2, *p* < 0.0001], suggesting highest scores in the young (*M* = 58.5, *SD* = 7.3) intermediate in the middle-aged (*M* = 54.2, *SD* = 8.3) and lowest in the oldest group (*M* = 39.1, *SD* = 9.8). *Post hoc* tests yielded substantial differences between all groups (all *p*-values < 0.0001). The re-test reliability of the total score of the trials 1 to 5 was 0.731 (*p* < 0.0001) in middle-aged and 0.572 (*p* < 0.0001) in the older group.

Also, recall of the interference list B (trial 6) showed significant group differences [*F*(2,518) = 180.5, *p* < 0.0001] with an decreasing number of correctly recalled items from young (*M* = 12.9; *SD* = 2.0) to middle aged (*M* = 11.8; *SD* = 2.5) to old subjects (*M* = 7.9; *SD* = 2.8). The groups differed significantly from each other (all *p*-values < 0.0001). The re-test reliability of the verbal fluency test was 0.298 (*p* < 0.05) in middle-aged and 0.190 (*p* < 0.05) in the older group.

The delayed recall of the list (trial 7) showed a similar pattern of decreasing performance with increasing age [*F*(2,518) = 151.5, *p* < 0.0001], indicating a larger number of correctly recalled items in young (*M* = 12.9; *SD* = 2.2) vs. middle-aged (*M* = 11.7; *SD* = 2.7) vs. old participants (*M* = 7.9; *SD* = 3.1). All groups significantly differed from each other (all *p*’s < 0.001). The re-test reliability of the verbal fluency test was 0.657 (*p* < 0.0001) in middle-aged and 0.662 (*p* < 0.0001) in the older group.

The recognition trial of 15 old and 15 similar new words revealed no differences between groups [*F*(2,518) = 1.0, *p* = 0.353]. This suggests that recognition of familiar items among new ones is the sole memory parameter unaffected by age. The re-test reliability of the verbal fluency test was 0.951 (*p* < 0.0001) in middle-aged and 0.458 (*p* < 0.0001) in the older group.

#### Multiple Choice Vocabulary Test (MWT-B)

**FIGURE 6:**
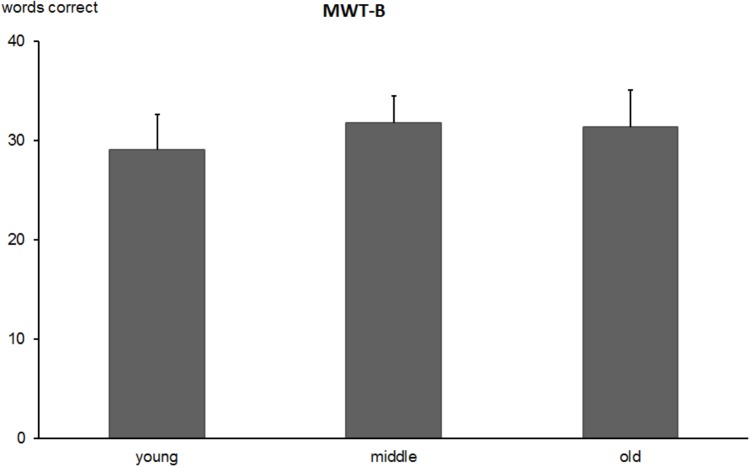
Total number of correctly marked words in Multiple Choice Vocabulary Test (MWT-B) in young, middle-aged, and old groups. Error bars reflect standard deviations.

The one-way ANOVA revealed an effect of age group [*F*(2,473) = 27.3, *p* < 0.0001], indicating a lower number of correct items in the young (*M* = 29.0, *SD* = 3.5) compared to middle-aged (*M* = 31.7, *SD* = 2.7) and old participants (*M* = 31.3, *SD* = 3.6). The descriptive results are presented in Figure 6. While the middle-aged and older groups did not differ from each other (*p* = 0.84) both older groups outperformed young participants (both *p* < 0.0001). The corresponding IQ-scores are 107.7 in young, 118.6 in middle-aged and 117.6 in old participants. The re-test reliability of the verbal fluency test was 0.727 (*p* < 0.0001) in the older group.

#### Digit-Symbol-Test

**FIGURE 7:**
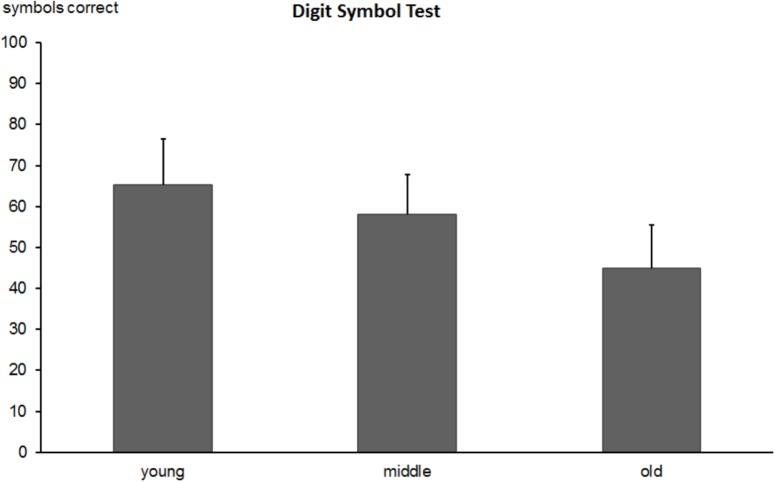
Total number of correctly produced symbols in the Digit-Symbol-Test (DST) in young, middle-aged, and old groups. Error bars reflect standard deviations.

The one-way ANOVA revealed significant group differences in the number of correctly filled symbols [*F*(2,518) = 164.5, *p* < 0.0001]. The number of correctly filled symbols decreased with increasing age (*M* = 65.1, *SD* = 11.2 vs. *M* = 57.9, *SD* = 9.7 vs. *M* = 44.9, *SD* = 10.6, for young, middle-aged, and old subjects, respectively; see Figure 7). *Post hoc* tests showed substantial differences between all groups (all *p* values < 0.0001). The re-test reliability of the digit-symbol-test was 0.666 (*p* < 0.0001) in middle-aged and 0.821 (*p* < 0.0001) in the older group.

#### d2 Test

**FIGURE 8:**
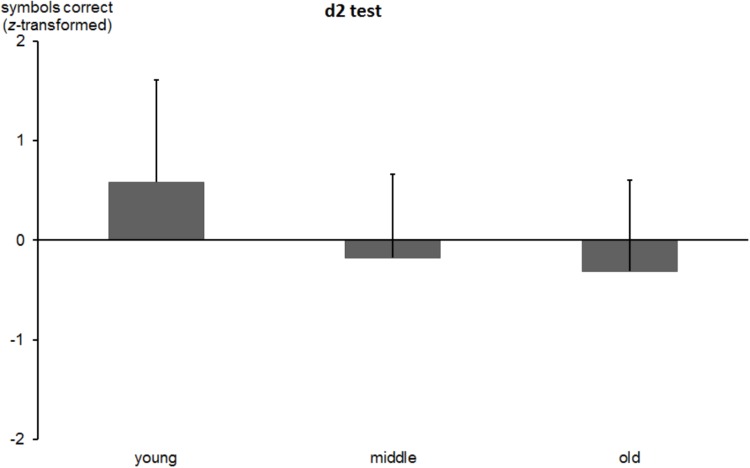
*z*-transformed number of correctly marked symbols in the d2 Test in young, middle-aged, and old groups. Error bars reflect standard deviations.

The one-way ANOVA indicated significant differences between age groups [*F*(2,521) = 44.9, *p* < 0.0001]. The *z*-transformed number of correctly crossed symbols decreased as a function of age (*M* = 0.58; *SD* = 1.02 vs. *M* = -0.17; *SD* = 0.83; vs. *M* = -0.31; *SD* = 0.91; for young, middle-aged, and old subjects, respectively; see Figure 8). Whereas the Bonferroni corrected *post hoc* comparison between middle-aged and old participants was non-significant (*p* = 0.458), the remaining differences were significant (all *p* values < 0.0001). The re-test reliability of the d2 Test was 0.799 (*p* < 0.0001) in middle-aged and 0.700 (*p* < 0.0001) in the older group.

#### Stroop Task

**FIGURE 9:**
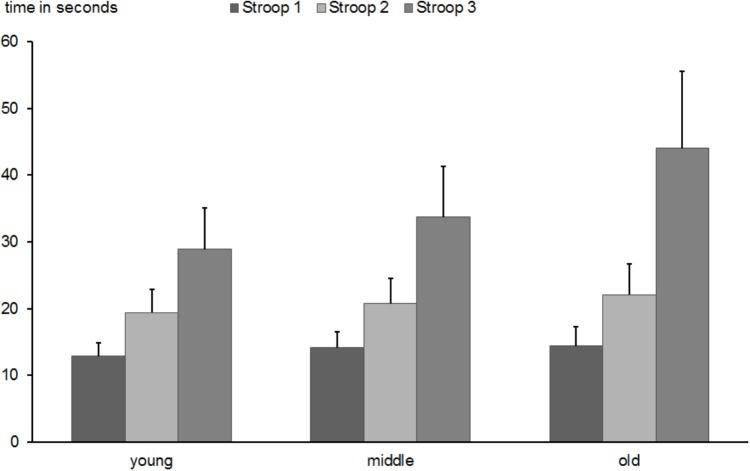
Mean time in seconds needed to conduct Stroop 1, 2, and 3 tasks in young, middle-aged, and old groups. Error bars reflect standard deviations.

The ANOVA with the factors task type and age group revealed main effects for task type [Stroop 1 vs. Stroop 2 vs. Stroop 3; *F*(2,1016) = 2819.3, *p* < 0.0001, $\eta_{p}^{2}$ = 0.847], age group [*F*(2,508) = 94.4, *p* < 0.0001, $\eta_{p}^{2}$ = 0.279], and a significant interaction of both factors [*F*(4,1016) = 116.5, *p* < 0.0001, $\eta_{p}^{2}$ = 0.314]. *Post hoc* examination revealed slower performance in Stroop 3 than in Stroop 2 and Stroop 1 (*M* = 36.0 s; *SD* = 10.9 vs. *M* = 20.8 s; *SD* = 4.2 vs. *M* = 13.8; *SD* = 2.5 s, all *p*-values < 0.0001). More importantly, whereas no group difference was found between middle-aged and older participants in the performance of Stroop 1 (*M* = 14.1 s; *SD* = 3.8; vs. *M* = 14.4 s; *SD* = 2.8, *p* > 0.05), substantial differences were found between young (*M* = 12.8 s; *SD* = 2.0) and middle-aged and young and old participants (*p-*values < 0.0001). Performance in the Stroop 2 task was reduced with increasing age (*M* = 19.4 s; *SD*: 3.4 vs. *M* = 20.8 s; *SD* = 3.7; vs. M = 22.0 s; *SD* = 4.2; for young, middle-aged, and old subjects, respectively; all *p*-values < 0.01). Interference processing measured by the Stroop 3 task strongly increased as a function of age (*M* = 28.8 s; *SD* = 6.2 vs. *M* = 33.6 s; *SD* = 7.6; vs. *M* = 44.0 s; *SD* = 11.6; for young, middle-aged, and old subjects, respectively; all *p*-values < 0.0001, see Figure 9). The re-test reliability of the Stroop 3 test was 0.754 (*p* < 0.0001) in the middle-aged and 0.627 (*p* < 0.0001) in the older group.

Finally, the interference effect assessed by the difference score between Stroop 3 and Stroop 2 was also substantially increased with age [*F*(2,508) = 134.7, *p* < 0.0001; *M* = 9.4 s; *SD* = 4.7 vs. *M* = 12.9 s; *SD* = 6.1; vs. *M* = 21.9 s; *SD* = 9.6; for young, middle-aged, and old subjects, respectively; all *p*-values < 0.0001).

#### Trail Making Test (TMT)

**FIGURE 10:**
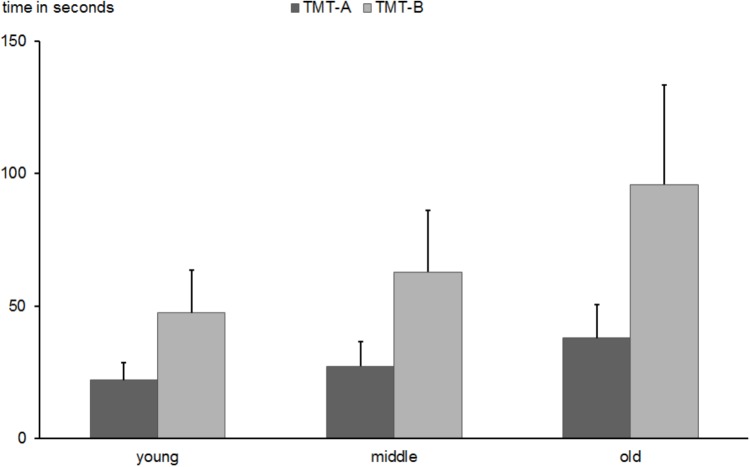
Mean time in seconds needed to conduct TMT-A and TMT-B tasks in young, middle-aged, and old groups. Error bars reflect standard deviations.

The ANOVA with the factors task type (TMT-A vs. TMT-B) and age group conducted for the TMT task revealed main effects of task type indicating longer performance time of the B than A version [68 s vs. 28 s; *F*(1,501) = 1395.4, *p* < 0.0001, $\eta_{p}^{2}$ = 0.736], age group [*F*(1,501) = 156.6, *p* < 0.0001, $\eta_{p}^{2}$ = 0.385], and an interaction between task type and age group [*F*(2,501) = 81.3, *p* < 0.0001, $\eta_{p}^{2}$ = 0.245]. The descriptive data are presented in Figure 10. *Post hoc* tests revealed age-related slowing in the TMT-A task (*M* = 21.9 s; *SD* = 6.5 vs. *M* = 27.1 s; *SD* = 9.2; vs. *M* = 37.8 s; *SD* = 12.6; for young, middle-aged, and old subjects, respectively; all *p*-values < 0.0001) as well as in the TMT-B task (*M* = 47.4 s; *SD* = 16.1 vs. *M* = 62.7 s; *SD* = 23.3; vs. *M* = 95.8 s; *SD* = 37.4; for young, middle-aged, and old subjects, respectively; all *p*-values < 0.0001). The re-test reliability of the TMT-B test was 0.381 (*p* < 0.01) in the middle-aged group. No data are available for the re-test of the old individuals.

In order to assess the effect of switching between task dimensions and to decompose the interaction, a difference score between TMT-B and TMT-A was computed and compared between age groups. A one-way ANOVA showed an increase in the switching ability as a function of age [*F*(2,501) = 81.3, *p* < 0.0001; *M* = 25.6 s; *SD* = 14.2 vs. *M* = 35.6 s; *SD* = 20.1; vs. *M* = 58.1 s; *SD* = 32.2; for young, middle-aged, and old subjects, respectively; all *p*-values < 0.0001].

### Correlation Analyses

Table 1 shows the results from the correlation analyses. In general, significant correlations between *n*-back performance and performance in other psychometric tests can be observed with regard to accuracy rather than speed of responses. Performance in the d2 Test correlates with *n*-back performance in the old group, whereas Trial Making Test and *n-*back performance are correlated consistently across all age groups. The pattern of significant relationships differs between age groups. Most strikingly, whereas *n*-back performance in young subjects shows the strongest correlation with performance in the TMT-B in speed and Stroop 3 in accuracy (switch and interference control), the middle-aged group exhibits correlations with digit span scores (short-term and working memory) and Stroop 1 and 2 performance (speed of processing) and performance in the TMT-B. In the old participants, correlations shift toward attentional and verbal memory capacities (Word Fluency Test, d2, Digit-Symbol-Test, Verbal Learning and Memory Test).

**Table 1:** Correlations between *n*-back performance (differences scores in response times and accuracy between 0-back and 2-back conditions) and performance measures of the different psychometric tests.

Age group	Young (*N* = 141)		Middle (*N* = 116)		Old (*N* = 163)	
			
Measure	RT	Acc	RT	Acc	RT	Acc
Psychometric task						
Digit Span Task (forward)	-0.093	-0.160	-0.054	**-0.356^∗∗^**	0.003	-0.153
Digit Span Task (backward)	-0.136	-0.201	-0.143	**-0.442^∗∗^**	-0.117	-0.157
Word Fluency Test	-0.076	-0.116	-0.013	-0.162	-0.179	-0.234^∗^
Digit Symbol Test	-0.251^∗^	-0.205	-0.235	-0.267^∗^	-0.211^∗^	**-0.302^∗∗^**
d2 Test (correct)	-0.205	-0.225^∗^	-0.067	-0.185	-0.154	**-0.265^∗∗^**
VLMT (Σ1-5)	-0.089	-0.100	-0.229	-0.160	0.082	-0.273^∗^
VLMT (recognition)	-0.073	0.014	-0.151	-0.187	0.047	-0.225^∗^
Stroop Task (part 1)	0.162	0.221^∗^	0.029	** 0.329^∗∗^**	0.024	0.056
Stroop Task (part 2)	0.197	0.237^∗^	-0.073	** 0.308^∗∗^**	0.149	0.141
Stroop Task (part 3)	0.210	** 0.286^∗∗^**	0.148	0.234^∗^	0.186	0.106
Trial Making Test (A)	0.261^∗^	0.236^∗^	-0.027	0.227	0.179	** 0.310^∗∗^**
Trial Making Test (B)	**0.338^∗∗^**	0.244^∗^	-0.051	** 0.390^∗∗^**	0.126	** 0.278^∗∗^**


*^∗^ Indicates correlations significant on an adjusted alpha level of *p* < 0.005 and bold ink indicates *p* < 0.001.*

Finally, we conducted a confirmatory correlation analysis using difference scores (incompatible–compatible) for accuracy from a computer-based Stroop task. Whereas the young group showed a correlation between the *n*-back and Stroop interference score in accuracy *r* = 0.235, *p* = 0.003. This association was attenuated in the middle-aged group *r* = 0.199, *p* = 0.007 and it was absent in older participants *r* = 0.139, *p* = 0.054.

## Discussion

In the present study, we examined performance of young, middle-aged, and older participants in the *n*-back task as well as in other cognitive tests in order to investigate changes in cognitive functions and particularly in WM across the lifespan. The second aim of the present study was to analyze which specific cognitive functions are-related to performance in the *n*-back task in young, middle-aged, and old participants to elucidate underlying mechanisms of WM decline in aging in more detail.

The first hypothesis, claiming that performance in the *n*-back task as well as in the wide range of fluid cognitive tasks decreases with increasing age, was confirmed, except for the delayed recognition in the VLMT and MWT-B representing measures of crystallized functions. The other psychometric tasks (2-back, Backward–Forward-DS, Digit-Symbol Test, Word Fluency, VLMT, d2, TMT and Stroop) were unambiguous and supported the hypothesis that fluid cognitive functions sustain a loss in performance with increasing age. More specifically, the underlying constructs, such as speed of processing (0-back, Stroop 1, Stroop 2, TMT-A), WMC (2-back, Backward-DS), short-term memory span (Forward-DS), verbal fluency, learning ability (VLMT Σ1-5), sustained and focused attention (d2, digit-symbol test, TMT-A), interference processing (Stroop 3), and switching ability (TMT-B) decrease with increasing age, which is already evident in middle aged subjects. It is important to note that the age-related decline in these tasks cannot be explained simply by a general reduction of processing speed with increasing age as some parameters, such as the WM measure (2-back–0-back), interference score (Stroop 3–Stroop 2) and switch costs (TMT-B–TMT-A), showed an age-related decline after removing individual differences in speed. These findings are in line with other results on age-related changes in executive functions (Salthouse, 1991, 2015; Van der Linden et al., 1994; Grégoire and Van der Linden, 1997; Braver and West, 2008; Basak and Verhaeghen, 2011; Bopp and Verhaeghen, 2018; Gajewski et al., 2018, but see Verhaeghen, 2011, 2014; Rey-Mermet and Gade, 2017; Rey-Mermet et al., 2017).

However, the most important results are those in relation to our second hypothesis: the correlations between *n*-back, performance and other psychometric tasks with respect to the different constructs as a function of age. This analysis sheds more light on specific age-related changes in cognitive strategies during 2-back performance. In particular, we investigated correlations between the performance in the *n*-back task and other psychometric tasks in each age group and expected that the cognitive functions that share variance with 2-back would correlate, whereas 2-back-performance should not correlate with measures of unrelated functions. The correlational analysis provided small to moderate but significant coefficients between 0.2 and 0.4 (see Schmiedek et al., 2014, for a discussion of possible reasons). One reason may be the used difference between 2-back and 0-back as a measure of WMC. More sensitive, however, might be pure RT and accuracy scores in each condition. Thus, the correlations in the present study reflect associations between different cognitive domains and a pure measure of WM capacity.

The results show that *n*-back performance in young participants shares variance mainly with executive functions, such as interference control (Stroop 3) in accuracy and task switching and updating (TMT-B) in speed. Middle-aged participants showed an association between 2-back performance in accuracy and measures of short-term and WM (Forward- and Backward-DS), speed of processing (Stroop 1 and 2) and task switching and updating (TMT-B), whereas older participants’ performance in the *n-*back accuracy was related to the d2, digit-symbol test and TMT-A, reflecting measures of attention and processing speed, different memory domains (VLMT), and task switching and updating (TMT-B). Thus, the results suggest that younger individuals involve mainly executive functions to perform the 2-back task, whereas in older subjects performance is associated primarily with attentional functions. Finally, the fact that TMT-B was correlated with *n*-back performance in each group suggests that task switching and updating is the common function that is consistently associated with this task across the life-span.

Although, as discussed above, the correlational analysis provided moderate coefficients, the finding was strengthened by the results of the confirmatory correlation analysis using interference scores from a computerized version of the Stroop test: while a substantial correlation between interference scores in accuracy of the Stroop test and *n*-back performance in accuracy was found in young subjects, this association was attenuated in the middle-aged group and it disappeared in old participants. These results allow some cautious inferences about functional mechanisms contributing to *n*-back performance in young, middle and older age. Firstly, the early decline of interference control assessed by Stroop 3 already apparent in the middle-age group (as illustrated in Figure 9) may require to compensate the deficient function by an another one. Some functions are not or at least to lesser extent subject of age-related decline (e.g., 0-back in Figure 2, Stroop 1 and 2 in Figure 9, or delayed recognition in Figure 5). The deficits in executive functions like interference processing used mainly in young age may be compensated by less vulnerable and less compromised cognitive functions by an involuntary strategy shift (Park and Reuter-Lorenz, 2009).

The substantial correlations between Stroop 3 and 2-back in young participants strengthen the findings of Kwong See and Ryan (1995), who showed that *n*-back shares more variance with Stroop than with STM tasks. These and our findings suggest that interference processing is crucial in both tasks 2-back and Stroop. This suggests that *n*-back involves interference processing: it seems plausible that inhibition of the recently stored item in trial *n*-1 and re-activation of the item from trial *n*-2 to compare it with the upcoming item in trial *n* is the crucial process involved in the task. Similarly, in the Stroop task, inhibitory control is essential to suppress the overlearned response to name the incongruent color. This is in line with the notion that WM and inhibitory control need one another and co-occur as whenever one goal is hold in mind irrelevant information has to be inhibited (Kane and Engle, 2003; Diamond, 2013). This is also in line with current models suggesting that *n-*back can be described as a paradigm that involves conflict processing (Rac-Lubashevsky and Kessler, 2016a,Rac-Lubashevsky and Kessler, 2016b).

Our findings are also consistent with age-related decline of executive functions described in the literature but the mechanisms underlying compensatory strategies to perform a task that requires executive control are still less understood (Turner and Spreng, 2012; Sala-Llonch et al., 2015). The results of the present study suggest that attentional resources, memory, maintenance of a goal, continuous switching, and updating are crucial processes relevant for successful performance in the *n*-back task in older age whereas interference control seems to reflect the most relevant domain in young individuals. It can therefore be speculated that broad processing resources are involved in compensating for executive deficits in older age, while young subjects use a few, but more efficient executive functions to perform the *n*-back task.

## Conclusion

In sum, our findings are consistent with previous results reporting age-related reduction in *n*-back performance and decline of the most fluid cognitive functions. This decline begins already in the midlife. A strong age-related impairment was observed in respect to executive functions that are crucial for successful performance in the *n-*back task. Correlational analyses conducted for each age group separately indicate an association between *n*-back performance and Stroop interference both in the paper pencil and the computerized version of the task in young individuals, whereas this association was attenuated or even absent in older age. Instead, other cognitive functions like attention, short and long-term memory were related to *n*-back performance in middle-aged and older age. The most consistent function related to *n-*back performance in all age groups was attentional switching and updating as measured by the TMT-B. The results of the present study indicate an age-related involuntary shift of processing strategies to successfully perform the *n*-back task and to compensate for deficits in interference control.

Taken together, the 2-back task is a complex cognitive task that measures a conglomerate of distinct cognitive functions that are differently involved depending on age. Further research is needed to extract the functional components in more detail.

## Author Contributions

PG designed the study, analyzed the data, and wrote the manuscript. EH analyzed the data and wrote the manuscript. MF designed the study and approved the final version of the manuscript. ST analyzed the data and wrote the manuscript. EW wrote the manuscript and approved the final version of the manuscript.

## Conflict of Interest Statement

The authors declare that the research was conducted in the absence of any commercial or financial relationships that could be construed as a potential conflict of interest.
